# Case of Hypertrophy of the Colon

**Published:** 1839-09-14

**Authors:** N. L. Thomas

**Affiliations:** Tennessee


*Case of Hypertrophy of the Colon.* By N. L. Thomas, M. D., of Tennessee.

To the Editors of the Medical Examiner.

Gentlemen,—In compliance with your request to contribute something to your valuable journal, I send you the following case, which is at your service. My attendance having been rather irregular, from the hopelessness of the case, the details are rendered somewhat incomplete.

Margaret, a negress, æt. 15, had been complaining for four years when I first saw her, 15th April. She was rather thin, somewhat under size, pulse normal, abdomen swelled, and subject to a remarkable degree of roaring, which could be heard at ten yards distance, and made her avoid going into company; the most considerable pressure, however, occasioned no pain. Bowels regular, and tongue clean; appetite weak; frequent colicky pains, with vomiting, particularly after eating, which made her very cautious in her diet. Mammæ considerably developed, but had never menstruated. By shampooing the abdomen for a minute, a great roaring commenced, and the transverse colon rose in bold relief above the other intestines, so as to be distinctly felt throughout nearly its whole course. These symptoms continued, with but little change, up to June 18th, she being able to perform moderate labour in the field. At the last named date, the vomiting increased so much, that stercoraceous matter was thrown up in considerable quantities. A dose of calomel and laudanum checked this symptom, but she never was able afterwards to perform her ordinary work, and was confined to the house till her death, which occurred August 29th. The only change that took place in the last six weeks of her life, was a diarrhœa, which could not be controlled, and gradually destroyed her. The treatment was mostly palliative after the first few weeks, and consisted principally of the preparations of opium with astringents and tonics.

*Autopsy.*—External appearance usual, except the abdomen, which was much fuller than is common after such protracted confinement; the contents of the chest normal, except an old and firm adhesion of the point of the lower lobe of the left lung to the diaphragm; the contents of the abdomen were natural, except the alimentary canal; the stomach healthy, but rather small, and the coats seemed rather thickened; small intestines but slightly diseased, but the *colon* was of a lead colour, devoid of cells, and of at least four times its usual dimensions, except at one or two points, where it was red externally, and so contracted as not to admit the end of the little finger; the mucous coat was of the colour of stewed prunes, and could be readily removed by the finger nails. Ulcerations were found nearly throughout its whole extent, and their disposition was extremely singular. They were so placed as to range exactly with the transverse and longitudinal bands that produce its cellular appearance, and had removed every trace of these bands, and at once explained the smooth appearance of the intestine. Not only was the cellular structure removed, but the gut was evidently very much elongated. The ulcers were from a very small size to more than that of a ninepenny-piece, and were deep and well marked. There was an evident hypertrophy of the whole gut. The ileocolic valve was very much contracted, and seemed to consist of numerous muscular fibres projecting into the intestine in a confused mass. The colon was filled with a large quantity of fluid, of the colour and consistence of yeast. I forgot to say that the cystic duct was impervious, which will account for the colour of the fluid spoken of,—and the gall bladder was much distended. The uterus was very small. The internal surface was white, and approached the firmness of cartilage. The ovaries were also very hard. The mesenteric glands were from the size of hazlenuts to that of plumbs. They were filled, as usual, with caseous and calcareous matter.

*Montgomery county*, *Tenn.*, Sept. 1st, 1839.

---

WASHINGTON CITY,

*August* 23*d*, 1839.

To the Editors of the Medical Examiner.

GENTLEMEN,—In the extract from the case of Margaret T., published in the Medical Examiner, (33d number,) my young friend, who had charge of the notes of the case, has omitted to present some of its most interesting features, viz:—1st. The monthly discharge of a bloody fluid from the stomach, resembling the catamenia, which continued till about 1st January, 1832,—the period when the catamenia resumed their natural course. 2d. The daily discharge of the urinous fluid from the same organ for several years, up to the period of her delivery of twins; and, lastly, the great length of time this poor creature could exist without having her urine taken from her—generally six weeks—and often two, even three months.

In presenting this case to the public, I am induced to call attention to the following inquiries, on which I shall be gratified to have your views, should you deem them of sufficient importance to occupy a small portion of your valuable time.

Are not the two first clearly illustrative of the assumption of the action of the uterus and kidneys, by the stomach? Were the kidneys in fault? Was the secretion, after reaching the bladder, absorbed and carried off by the stomach and skin, not having a vent by the natural passage? In this event, how did it reach the stomach? By what agency were the convulsions, which were decidedly epilectic, produced? If by the irritation of the catheter, why did they continue so long after this source of irritation ceased to exist? We have often seen syncope produced by the introduction of the catheter, even convulsive twitchings,—but they would subside immediately upon its being withdrawn.

These convulsions resisted the most active remedial measures; nothing that we could devise seemed to produce the slightest amelioration of the attacks. Large bleedings, antispasmodics in very large doses, &c. &c., were resorted to both in anticipation and during the attacks, without effect.

Very respectfully, your ob't servant,
THOS. MILLER.

[There can be little doubt that there was a true vicarious menstruation—an assumption of the functions of the uterus by the stomach, which is by no means an unusual occurrence with women affected with certain forms of irregular menstruation.

It is much to be regretted that the occasion was not taken advantage of, for ascertaining whether the urine was really absorbed after having passed into the bladder. This is certainly against ordinary observation, and we should admit much more readily that the kidneys had, for a time, ceased their action, and that the urea was eliminated from the blood by the skin and stomach. If the patient had been more constantly under observation, it could have been known with certainty whether the urine was ever accumulated in large quantity in the bladder. The question, of course, cannot now be decided.

The convulsions we should regard as hysterical, and not strictly epileptic. That is, the irritation of the bladder and uterus was carried to a much greater degree than usual, and gave rise to intense and prolonged convulsions, assuming the epileptic form. This is certainly a most unusual symptom.

That the case was one strictly dependent upon uterine irritation, is very clear from the subsidence of the symptoms after child-bearing. It is another of those anomalous forms of disease to which we apply the term hysteria, and which are at least curious from their developing many obscure physiological relations.—Eds.]

